# Independent control of electrical and heat conduction by nanostructure designing for Si-based thermoelectric materials

**DOI:** 10.1038/srep22838

**Published:** 2016-03-14

**Authors:** Shuto Yamasaka, Kentaro Watanabe, Shunya Sakane, Shotaro Takeuchi, Akira Sakai, Kentarou Sawano, Yoshiaki Nakamura

**Affiliations:** 1 Graduate School of Engineering Science, Osaka University, 1–3 Machikaneyama-cho, Toyonaka, Osaka 560–8531, Japan; 2 Advanced Research Laboratories, Tokyo City University, 8–15–1 Todoroki, Setagaya, Tokyo 158–0082, Japan

## Abstract

The high electrical and drastically-low thermal conductivities, a vital goal for high performance thermoelectric (TE) materials, are achieved in Si-based nanoarchitecture composed of Si channel layers and epitaxial Ge nanodots (NDs) with ultrahigh areal density (~10^12^ cm^−2^). In this nanoarchitecture, the ultrasmall NDs and Si channel layers play roles of phonon scattering sources and electrical conduction channels, respectively. Electron conductivity in* n*-type nanoacrhitecture shows high values comparable to those of epitaxial Si films despite the existence of epitaxial NDs. This is because Ge NDs mainly scattered not electrons but phonons selectively, which could be attributed to the small conduction band offset at the epitaxially-grown Si/Ge interface and high transmission probability through stacking faults. These results demonstrate an independent control of thermal and electrical conduction for phonon-glass electron-crystal TE materials by nanostructure designing and the energetic and structural interface control.

Energy and environmental issues have motivated studies on thermoelectric materials which convert wasted heat into electricity^1^. Therein, the dimensionless figure of merit strongly related to the energy conversion efficiency, has been focused on in this research field, and it is written as *ZT* = *S*^2^*σT*/**κ**, where *S* is Seebeck coefficient, *σ* is electrical conductivity, **κ** is thermal conductivity, and *T* is absolute temperature^2^. Based on the concept of phonon-glass electron-crystal^3^, high *ZT* value is accomplished by increasing *σ* values and reducing **κ** values concurrently. However, the values of thermoelectric properties are correlated, and as a result, it has been difficult to control each value independently. Therefore, it has been a vital goal for long. So far, limited materials with relatively high *σ* and low **κ**, such as chalcogenide^4^,^5^ have been used in practical use. Such materials include rare and toxic elements, which hinders the use of thermoelectric materials in the wide range of field. Recently, a lot of studies on *ZT* value enhancement by nanostructuring has drawn much attention, where a main aim is to reduce **κ** while maintaining high *σ* value by utilizing the mean free path differences between phonons and carriers^6^,^7^. However, **κ** reduction while maintaining high *σ* is still difficult in conventional nanostructuring approach.

Si-based thermoelectric materials are expected to be developed because Si is ubiquitous and non-toxic materials established in industry^8^,^9^. Bulk Si has large power factor *S*^2^*σ*, but also exhibits large **κ**, resulting in low *ZT* value. Therefore, nanostructuring for **κ** reduction while keeping high *σ* is promising for realization of Si-based thermoelectric materials^10^,^11^,^12^,^13^. There have been many studies for developing Si-based nanostructured thermoelectric materials. Nanostructured bulk Si and SiGe bulk alloys made by ball-milling and sintering were intensively studied, leading to **κ** reduction^11^,^14^,^15^,^16^. However, limited crystal size resulted in limited **κ** reduction (~2–12 Wm^−1 ^K^−1^) and their random crystal orientation led to *σ* degradation. Therefore, a new methodology for **κ** reduction and high *σ* maintenance using nanostructuring is expected to be established.

We have presented two kinds of the nanoarchitectures based on the following strategy^17^,^18^,^19^. One is “coherently connected Si nanocrystals (NCs)”, where ultrasmall Si NCs (~3 nm) are covered with 1-monolayer (1-ML) thick SiO_2_ film and their crystallographic orientations are uniformed by connecting neighbouring NCs through Si nanowindows (<1 nm) in the ultrathin SiO_2_ films. In this nanostructure, nanometer scale rough interfaces formed by NCs lead to the large phonon scattering and coherent carrier wave function is considered to form in NCs. Actually, the drastic **κ** reduction from bulk Si value down to ~0.8 Wm^−1^ K^−1^ at *T* = 300 K was realized^17^, which was attributed to the large interfacial thermal resistance due to the phonon scattering at the ultrasmall Si NC interfaces^17^. To keep bulk-like *σ* in Si while using the NCs as phonon scattering sources, we proposed another nanoarchitecture which stacks alternately epitaxial Ge nanodots (NDs) with ultrahigh areal density (~10^12^ cm^−2^) for phonon scattering sources and epitaxial Si layer for carrier conduction layers^18^,^19^. In this nanoarchitecture, we expected that phonons are scattered by the ultrasmall Ge NDs, whereas carrier conduction is smoothly done in epitaxial Si layers including coherently-embedded Ge NDs. Recently, we have actually fabricated this nanoarchitecture^18^, as shown in Fig. 1a and demonstrated the large **κ** reduction to 1.2 Wm^−1 ^K^−1^, which is the smallest value in SiGe system at the smaller Ge content (<~15%)^19^. In this nanoarchitecture under non-doping condition, the **κ** reduction mechanism was revealed^19^. The phonon transport/scattering in this nanoarchitecture is analogous to light wave scattering theory by nanoparticles (Rayleigh or Mie scattering) including the ND shape effects^19^. On the other hand, the carrier transport and thermoelectric power in our proposed nanoarchitecture have not been investigated. Also, the thermal conduction of highly-doped samples should be examined because it is more important in terms of practical use. Now, it is expected to demonstrate independent control of **κ** and *σ* by clarifying the carrier and phonon transports, and thermoelectric power in the nanoarchitecture.

In this paper, the feasibility of independent control of **κ** and *σ*, which has been a vital goal, is demonstrated in the nanoarchitecture of Si films including epitaxial NDs. Notably, drastic reduction of thermal conductivity is demonstrated in doped-nanoarchitecture beyond non-doped sample cases, and simultaneously electron conductivity in *n*-type nanoarchitectures exhibits the high value comparable to that of epitaxial films of Si, the bulk of which has high electrical conductivity. These results demonstrate that this nanoarchitecture is a potential phonon-glass electron-crystal material.

## Methods

**Figure 1:**
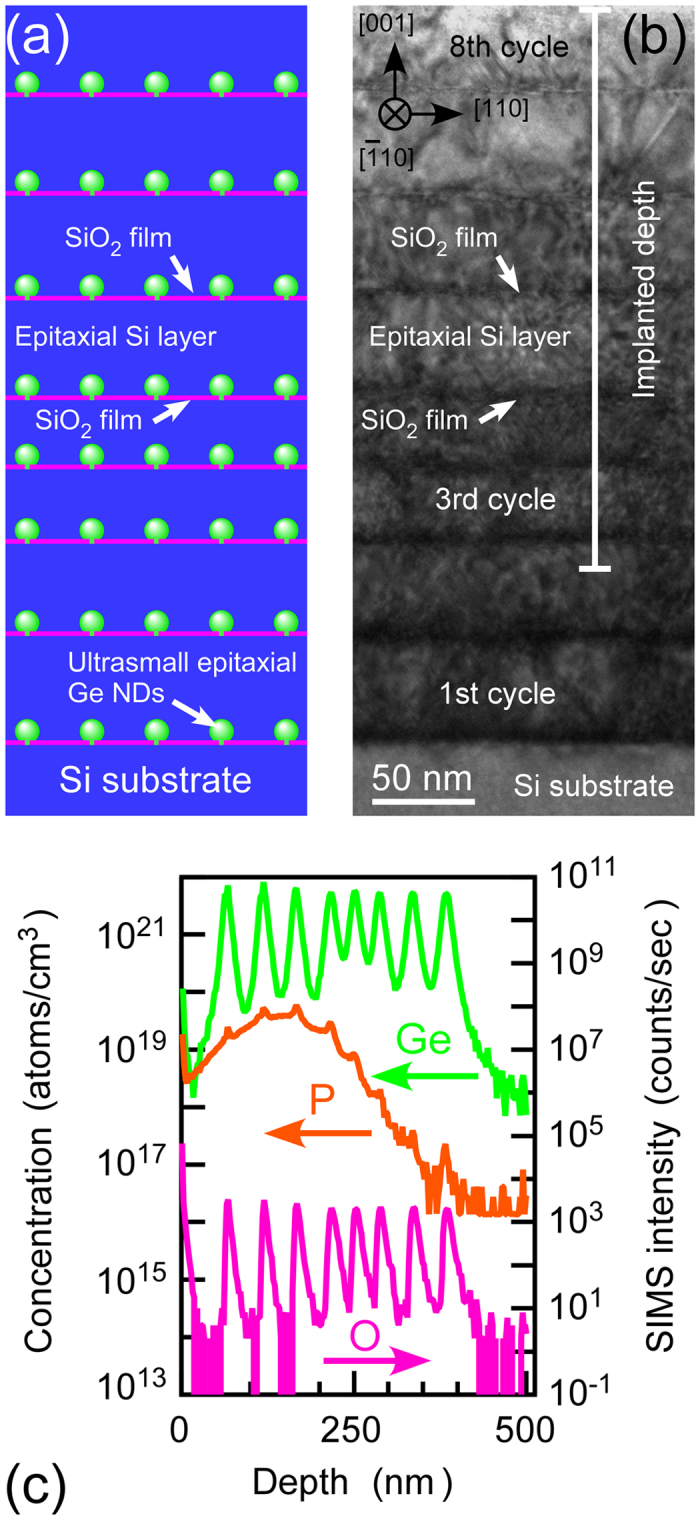
(**a**) Schematic of our proposed nanoarchtecture; namely stacked structure of epitaxial Ge NDs and Si layers. (**b**) Cross-sectional TEM image of the typical stacked structure (8-nm NDs/376-ML Si sample) after P ion implantation (110 keV, 4 × 10^14^ cm^−2^). Electron beam energy is 200 keV. (**c**) SIMS profiles of the stacked structure shown in (**b**).

Non-doped Si(001) substrates were introduced into a molecular beam epitaxy (MBE) chamber with a reflection high energy electron diffraction (RHEED) apparatus at a base pressure of ~3 × 10^−8^ Pa after conventional wet chemical treatment. After degassing the substrates at 500 °C for several hours, 100-nm-thick Si buffer layers are epitaxially grown on Si substrates at 500 °C to obtain clean Si(001)-(2 × 1) surfaces. The clean Si surfaces were then oxidized at 500 °C for 10 min at an oxygen pressure of 2 × 10^−4^ Pa to form ultrathin (<1 nm) SiO_2_ films^20^,^21^,^22^. Ge of 7–20 MLs was deposited on the ultrathin SiO_2_ films at 500 °C to form epitaxial Ge NDs, which detail is written in our previous papers^23^,^24^,^25^. Si of 50–376 ML was deposited on the Ge NDs at 400 °C to form epitaxial Si layers. The Si layers were then oxidized to form ultrathin (<1 nm) SiO_2_ films at 450 °C. The ultrathin SiO_2_ films/Ge NDs/Si layer structure formed using the above three processes was defined as “one cycle structure” in this paper. This cycle was repeated eight times to form eight cycle structures of Si/Ge NDs, as shown schematically in Fig. 1a. In this paper, we refer to the stacked structure of Ge NDs with *x*-nm diameter and *y*-ML Si layer as “*x*-nm NDs/*y*-ML Si” sample. By RHEED observations at every growth stage, it was confirmed that stacked structures of Ge NDs and Si layers were epitaxially grown on Si substrates. This epitaxial growth mechanism in the stacked structure is reported in our previous study^18^. For carrier doping into the nanoarchitectures, ion implantation was performed using P and BF_2_ ions with kinetic energy of 25–160 keV at dose concentration of 4 × 10^14^−1 × 10^15^ cm^−2^, followed by activation annealing: rapid thermal annealing at temperatures of 635 °C in nitrogen gas at atmospheric pressure.

Secondary ion mass spectrometry (SIMS) is carried out to investigate the surface-depth profiles of Ge, O and dopant atoms using 2–5 keV Cs^+^ ion. Surface-depth profiles of dopant atoms implanted at different primary ion energies are calculated by SRIM program^26^, demonstrating a consistency with SIMS surface-depth profile of implanted P atoms, which detail is written in Fig. S1 in Supplementary Information. In-plane electrical conductivities *σ* and Hall mobilities *μ*_H_ are measured by Hall effect measurement in van der Pauw method. Thermal conductivity measurements were carried out by 2ω method at room temperature, where the 100-nm Au films were formed on the surfaces of the stacked structures for the heating electrodes. Thermoreflectance of the Au films on the stacked structures was measured using laser light (wavelength of 635 nm). Electrical resistances of doped stacked structures were confirmed to be much larger than that of Au film electrodes. The details of 2ω method were described in our previous papers^17^,^19^,^27^.

## Results and Discussion

Figure 1b shows cross-sectional high resolution transmission electron microscope (HRTEM) image of the stacked structure (8-nm NDs/376-ML Si sample) after P-ion implantation which corresponds to a schematic in Fig. 1a. This low magnification cross-sectional HRTEM image of the entire nanoarchitecture shows the periodic structures characterized by straight line contrasts pointed by the arrows revealing the stacked structures. These straight line contrasts are considered to be the ultrathin SiO_2_ films and the layers between these line contrasts are Si layers. Figure 1c shows SIMS profiles of the stacked structure of the sample shown in Fig. 1b. Ge and O depth-profiles exhibit eight peaks responsible for eight cycle structure in Fig. 1a, and the positions of these peaks also correspond to those of the ultrathin SiO_2_ film layers in Fig. 1b. The consistency of Ge and O peak positions implied that Ge NDs existed on the ultrathin SiO_2_ films. These results indicated that the stacked structures were kept after the carrier doping.

Here, we discuss the P surface-depth profiles in the nanoarchitecture after dopant implantation (primary energy of 110 keV). P surface-depth profile agreed with the broad Gaussian profile calculated by SRIM (standard deviation of 50.3 nm and peak position of 147 nm in depth) except for eight small sharp peaks. The positions of the eight small sharp peaks in P surface-depth profile measured by SIMS correspond to those of O peaks in SIMS related to the ultrathin SiO_2_ films, indicating that some of P atoms were trapped near the ultrathin SiO_2_ interfaces. The broad Gaussian profile of implanted P atoms indicated an implanted depth of 276 nm, in which region 99.5% of doped carriers existed (See Fig. S1 in Supplementary Information). This depth can be considered to be carrier conduction layer thickness. This indicates that implanted P does not reach Si substrate (See Fig. S1 in Supplementary information). The surface-depth profiles of implanted B were also calculated by SRIM in a similar way to determine the implanted depth. This also showed that implanted B does not reach Si substrate. Electrical sheet resistances of the *n*- and *p*-type nanoarchitectures were confirmed to be much smaller than that of non-doped Si substrate, supporting that the thermoelectric measurements of the stacked structure in the present work probes that of stacked structures without substrate property.

**Figure 2:**
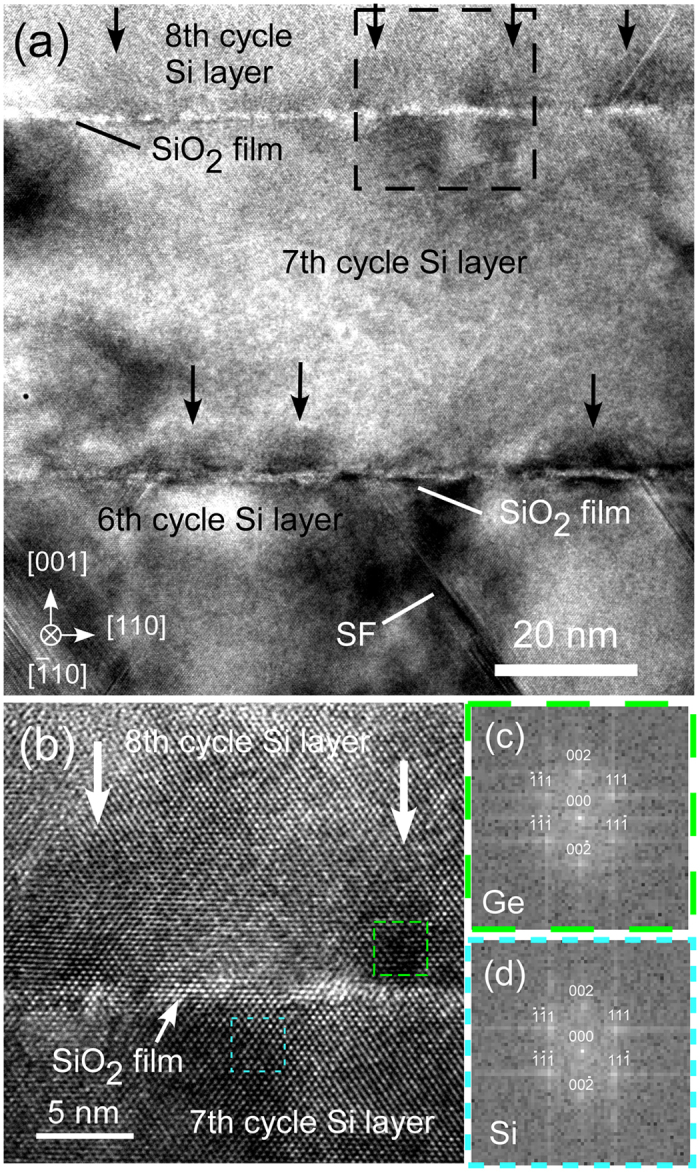
(**a**) Cross-sectional HRTEM image of the P ion-implanted (110 keV, 4 × 10^14^ cm^−2^) stacked structure (8-nm NDs/376-ML Si sample) at higher magnification. The electron beam energy is 200 keV. (**b**) Enlarged image of the boundary between 8th and 7th cycle Si layers marked by the dashed square in (**a**). (**c**,**d**) FFT patterns of the regions marked by dashed squares in (**b**).

Figure 2a shows a cross-sectional HRTEM image around Si layer in 7th cycle structure (7th cycle Si layer) in the P-doped *n*-type nanoarchitecture. It reveals that ultrasmall Ge NDs with relatively black contrasts (black arrows) exist on SiO_2_ films in the nanocarchitecture. A lot of stacking faults were also observed as denoted by a mark of “SF”. Figure 2b shows a high magnification cross-sectional HRTEM image of the dashed square region in Fig. 2a. Figure 2c,d are the fast Fourier transform (FFT) patterns of the areas marked by dashed squares in Fig. 2b, which correspond to Ge ND regions and 7th cycle Si layer, respectively. FFT analyses demonstrated that the crystal structures of Si layers and Ge and their epitaxial growth were not destroyed after carrier doping. In addition, it was found that Si layers were strain-relaxed above Ge NDs within a typical distance of ~1 nm (See Fig. S4 in Supplementary Information). This relaxation could be attributed to three dimensional spherical shape of ultrasmall NDs and to the small contact area between Si and underlying ultrasmall Ge NDs on the ultrathin SiO_2_ films, where Ge NDs have small coverage of 40%^19^. This is totally different from Stranski-Krastanov Ge NDs with wetting layer^19^,^28^.

**Figure 3:**
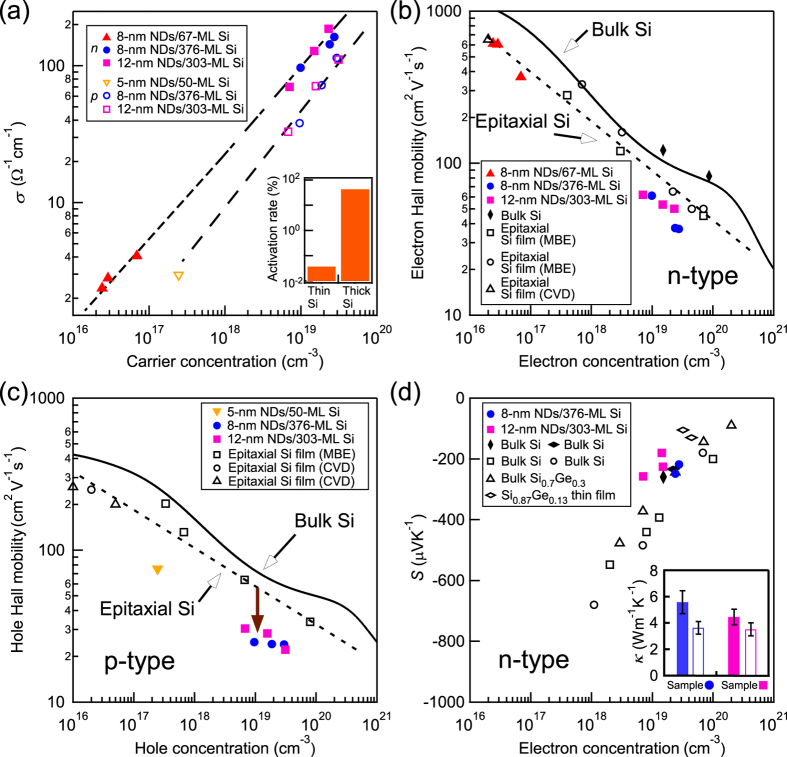
(**a**) Electrical conductivities of our nanoarchitectures. The solid and open marks indicate P- and B-doped nanoarchitectures, which exhibited *n*-type and *p*-type conductivities, respectively. The dashed lines are denoted for eye-guide. The inset shows the carrier activation rate of nanoarchitectures in the case of thin (~70 ML) and thick (>300 ML) Si layers. (**b**) Hall mobilities of P-doped (*n*-type) and (**c**) B-doped (*p*-type) nanoarchitectures, respectively. The solid marks denote our studies. The open marks indicate the values of reported epitaxial Si films on Si substrate with the dashed lines for eye-guide. In (**b**), open square, circle and triangle marks indicate the reported values of MBE^39^,^40^ and CVD^41^, respectively. In (**c**), the open square, circle and triangle marks indicate the reported values of MBE^40^ and CVD^42^,^43^, respectively. The solid lines are those of bulk Si calculated by empirical formula^44^. (**d**) Seebeck coefficients of P-doped typical stacked structure (8-nm NDs/376-ML Si sample and 12-nm NDs/303-ML Si sample). Open square, circle, triangle and diamond marks indicate the reported values of bulk Si 9,45, Si_0.7_Ge_0.3_ 45 and Si_0.87_Ge_0.13_ thin film^46^ for reference. The inset shows the thermal conductivities of non-doped (solid bar) and doped (open bar) nanoarchitectures. Dose condition is summarized in Table SI and SII in Supplementary Information. In the same samples, the larger dose causes the larger carrier concentration.

Figure 3 displays the carrier concentration dependence of the thermoelectric properties for P- and B-doped nanoarchitectures at room temperature. Hall effect measurements revealed that the P- and B-doped nanoarchitectures exhibited *n*-type and *p*-type conductivities, respectively. Figure 3a exhibits that the *σ* increases with carrier concentration in wide concentration range for both *n*-type (solid marks) and *p*-type nanoarchitectures (open marks), which is a similar tendency to that of bulk Si. It was found that in the case that Si layer in each cycle structure is thin, carrier concentration is smaller under the same dose condition: dopants are deactivated electrically. The inset shows carrier activation rates in *n*-type nanoarchitectures at the dose of 4 × 10^14^ cm^−2^, which were evaluated from SIMS and Hall effect measurements. While activation rate reaches of ~50% in the nanoarchitectures with thick Si layers (>300 ML), it is only 0.04% in those with thin Si layers (~70 ML). Low activation rate of nanoarchitectures with thin Si layer is attributed to the trap of dopant atoms at the Si/Ge NDs/SiO_2_ interfaces^29^.

In order to discuss the carrier transport in the stacked structures explicitly, we evaluated the *μ*_H_ values at both *n*- and *p-* type conductivities as shown in Fig. 3b,c. The values of the epitaxial Si films on Si substrate formed by MBE and chemical vapour deposition (CVD) were plotted for reference there. Electron Hall mobilities in *n*-type nanoarchitectures, *μ*_He_, are found to be almost equivalent to those of reported Si epitaxial films. This proved that ultrahigh density Ge NDs working as phonon scattering sources^19^ do not degrade *μ*_He_. This demonstrates a **κ** reduction (0.04– 0.008 times from that of bulk Si^19^) and retaining *σ* values.

We evaluated Seebeck coefficients of typical *n*-type nanoarchitectures as shown in Fig. 3d. The Seebeck coefficients agree with reported values of bulk Si, bulk SiGe and SiGe thin film. This means that the present nanostructuring does not affect Seebeck coefficient significantly. Next, we also evaluated the **κ** values of doped stacked structures. In our previous work, **κ** values of non-doped samples were useful for clarifying the phonon scattering mechanism related to **κ** reduction^19^. However, the *κ* measurement of doped-samples (10^19^−10^20^ cm^−3^) is important in terms of practical use. Thus, the *κ* values of the P-doped samples at carrier concentration of 2−3 × 10^19^ cm^−3^ (8-nm NDs/376-ML Si sample and 12-nm NDs/303-ML Si sample) were measured to be 3.6 and 3.5 Wm^−1^ K^−1^ respectively in direction perpendicular to the surfaces. This indicated that the drastic *κ* reduction was also confirmed in the case of doped nanoarchitecture. It should be noted that the *κ* values of “doped samples” showed about 20–40% smaller than those of non-doped stacked structure (~5.6 and ~4.5 Wm^−1^ K^−1^)^19^ as shown in inset in Fig. 3d. This is consistent with reported results of *κ* reduction by doping^11^,^30^. Then, this is considered by increase of wavelength range of scattered phonons due to addition of dopant atoms (impurity atoms) because phonons wavelength scattered by ten and several nm size NDs and by dopant atoms are considered to be different. Thus, our nanoarchitecture demonstrated the independent control of *σ* and *κ* and its effectiveness as thermoelectric materials sufficiently.

Here, we briefly discuss possible mechanisms of the independent control. We considered the band alignment between Ge NDs and Si layer. In *n*-type nanoarchitecture (electron concentration range of 10^16^–10^19^ cm^−3^), conduction band offset at the Si/Ge ND interface is small (~50 meV) due to the small gap of electron affinity, 4.0 eV for Ge and 4.05 eV for Si^31^, respectively. Recently, the similar small band offset effect was reported about *p*-type PbS with endotaxially introduced CdS^32^, where the suppression of carrier scattering at the interface was achieved because of the small valence band offset of ~0.13 eV at the interface of PbS/CdS. Therefore, in our present study, there is a possibility that the electron scattering at the interface can be suppressed at the Si/Ge ND interface with small band offset of ~50 meV. This value is comparable to the electron thermal energy, *k*_B_*T* at room temperature (approximately 30 meV). In addition, although our nanoarchitecture includes lots of stacking faults shown in Fig. 2a, the carrier electron scattering at stacking faults in Si (Fig. 2a) is found to be small by considering the high transmittance of electrons (~ 0.7) with *k*_B_*T* at room temperature^33^. Thus, Ge NDs work as scatterers not for electrons and but for phonons effectively.

Here, we briefly discuss the influence of the structural anisotropy in our nanoarchitecture on its TE properties. In the Si/Ge superlattice structure where flat Si and Ge layers are stacked alternately, the electrical anisotropy in *σ* was reported due to the strong structural anisotropy^34^. On the other hand, the isotropic TE properties were reported in the nanostructured bulk alloys with isotropic structure^35^. In the case that Si layer in one cycle structure in our nanoarchitecture is thin (~70 ML), TE properties could be expected to be isotropic ones because Ge ND positions become random due to the rough Si layers (See Fig. S3 in Supplementary Information) and thus the structure is quasi-isotropic. On the other hand, in the case of ordered stacked structures where Si layer is thick (>300 ML) as shown in Fig. 1b, one might assume that TE properties are anisotropic as is the case with the superlattice structure of Si/Ge flat layers^34^. However, *μ*_He_ values in both cases of thin and thick Si layers are almost the same as those of the epitaxial Si films without Ge NDs as shown in Fig. 3b, indicating that electrical conduction anisotropy caused by Ge ND existence is very weak. The lack of electrical conduction anisotropy can be explained by the weak influence of Ge NDs on the electron conduction in this nanoarchitecture, which is consistent with the above-mentioned weak electron scattering at Si/Ge ND interfaces.

Note that the *σ* values in *p*-type nanoarchitectures are lower than those in *n*-type ones in Fig. 3a. This is because of hole Hall mobility (*μ*_Hh_) degradation in *p*-type nanoarchitecture as shown in Fig. 3c. The *μ*_Hh_ values are much smaller than reported values of Si epitaxial films. The *μ*_Hh_ degradation by nanoarchitecturing is not elucidated experimentally at this stage although it could be associated with larger valence band offset than that in *n*-type stacked structures. We speculated another possible explanation: holes are scattered at the stacking faults with higher possibility than electron. It was reported that the *μ*_Hh_ value of *p*-type polycrystalline Si thin film is lower than that of *p*-type single crystalline bulk Si, and this mobility reduction is larger than that of *μ*_He_ value in *n*-type structures^11^,^36^. Therefore, there is a possibility that scattering probability of holes at the interfaces such as stacking faults in Si is higher than that of electrons intrinsically. Further studies are required to reveal origins of hole scattering and to improve the hole mobility of *p*-type nanoarchitectures.

Here we estimated the *ZT* value of this nanoarchitecture to discuss its thermoelectric performance. We focused on the sample with small Ge fraction (<3–5%) as typical one because the large amount use of rare metal Ge was not preferable. The rigorous *ZT* value is difficult to be estimated because the measured directions in *κ* and *σ* are different in this study. When considering the weak anisotropy of electrical conduction in the nanoarchitecture, the *ZT* estimation from these *κ* and σ can be a rough indication for the effectiveness of the nature of our nanoarchitecture like a reported paper^37^. The *ZT* value of our *n*-type nanoarchitecture was estimated to be ~0.07 at room temperature, using our results: σ = 162 Ω^−1^ cm^−1^, *κ* = 3.6 Wm^−1^ K^−1^, and |*S|* = 220 *μ*VK^−1^ of our 8-nm NDs/376-ML Si sample (Ge content ~3%) at electron concentration of ~3 × 10^19^ cm^−3^. This estimated *ZT* value is comparable to high value at room temperature in nanostructured bulk Si with small Ge content of ~3% under the optimized doping condition (~1−2 × 10^20 ^cm^−3^) in the previous work^15^. However, our nanoarchitecture is not optimized yet, and there is a room of further enhancement of *ZT* by optimizing doping conditions and/or other structural factors. For example, the *ZT* of 0.2 at room temperature in our nanoarchitecture can be expected by optimizing the electron concentration of 1−2 × 10^20^ cm^−3^ that is an optimized value in bulk Si case, assuming that our sample had the same power-factor-dependence on the electron concentration of that of bulk Si. So far, this *ZT* is the largest value in bulky Si materials including small Ge (<3–5%)^15^,^38^. These results demonstrate the possibility of realization of Si-based TE materials.

## Conclusions

We investigated thermoelectric properties of Si-based epitaxial Ge NDs nanoarchitectures after *ex-situ* doping. The electron mobilities of *n*-type nanoarchitectures were comparable with those of epitaxial Si films, unlike hole mobility in *p*-type nanoarchitectures. The value of Seebeck coefficient of typical *n*-type sample was comparable with the reported values of bulk Si and bulk SiGe. Thermal conductivity *κ* of doped-samples showed drastic *κ* reduction where *κ* values are around 20–40% smaller than non-doped sample cases. These result demonstrated that the feasibility of independent control of σ and *κ* by nanostructure designing for a phonon-glass electron-crystal TE material.

## Additional Information

**How to cite this article**: Yamasaka, S. *et al.* Independent control of electrical and heat conduction by nanostructure designing for Si-based thermoelectric materials. *Sci. Rep.* **6**, 22838; doi: 10.1038/srep22838 (2016).

## Supplementary Material

Supplementary Information
