# Supplementary material for: Independent control of electrical and heat conduction by nanostructure designing for Si-based thermoelectric materials
Source: Sci Rep. 2016 Mar 14;6:22838. doi: 10.1038/srep22838 (PMC4789645; doi:10.1038/srep22838)
Supplement: Supplementary Information [file srep22838-s1.pdf]

Supplementary Information

Independent control of electrical and heat conduction by nanostructure designing for Si-based thermoelectric materials

Shuto Yamasaka[1], Kentaro Watanabe[1], Shunya Sakane[1], Shotaro Takeuchi[1], Akira Sakai[1], Kentarou Sawano[2], Yoshiaki Nakamura[1,*]

[1] Graduate School of Engineering Science, Osaka University, 1-3 Machikaneyama-cho, Toyonaka, Osaka 560-8531, Japan

[2] Advanced Research Laboratories, Tokyo City University, 8-15-1 Todoroki, Setagaya, Tokyo 158-0082, Japan

*Corresponding author

E-mail: nakamura@ee.es.osaka-u.ac.jp

I. Sample information

Table SI Sample information of *n*-doped stacked structures.

ID	Structure	Film Thickness (nm)	Ion energy (keV)	Ion dose (cm^{-2})	Projected range (nm)	Standard deviation (nm)	Implanted depth (nm)
1	8-nm NDs/ 67-ML Si	85	25	4×10^{14}	36.4	16.1	78
2	8-nm NDs/ 67-ML Si	85	25	7×10^{14}	36.4	16.1	78
3	8-nm NDs/ 67-ML Si	85	25	1×10^{15}	36.4	16.1	78
4	8-nm NDs/ 376-ML Si	422	110	4×10^{14}	147	50.3	276
5	8-nm NDs/ 376-ML Si	422	110	7×10^{14}	147	50.3	276
6	8-nm NDs/ 376-ML Si	422	110	1×10^{15}	147	50.3	276
7	12-nm NDs/ 303-ML Si	353	110	4×10^{14}	147	50.3	276
8	12-nm NDs/ 303-ML Si	353	110	7×10^{14}	147	50.3	276
9	12-nm NDs/ 303-ML Si	353	110	1×10^{15}	147	50.3	276

Table SII Sample information of *p*-doped stacked structures.

ID	Structure	Film Thickness (nm)	Ion energy (keV)	Ion dose (cm^{-2})	Projected range (nm)	Standard deviation (nm)	Implanted depth (nm)
10	5-nm NDs/ 50-ML Si	63	25	1×10^{15}	28	11.1	56.8
11	8-nm NDs/ 376-ML Si	422	160	4×10^{14}	149	45.6	267
12	8-nm NDs/ 376-ML Si	422	160	7×10^{14}	149	45.6	267
13	8-nm NDs/ 376-ML Si	422	160	1×10^{15}	149	45.6	267
14	12-nm NDs/ 303-ML Si	353	160	4×10^{14}	149	45.6	267
15	12-nm NDs/ 303-ML Si	353	160	7×10^{14}	149	45.6	267
16	12-nm NDs/ 303-ML Si	353	160	1×10^{15}	149	45.6	267

II. Dopant surface-depth profiles, carrier profiles, and activation rate

The surface-depth profile of dopants is shown in Fig. S1(a). We find little difference between calculation and SIMS result except for P small sharp peaks in SIMS. This suggests that SRIM calculation is applicable to these nanostructures implanted at different primary ion energies and that P diffusion during the rapid thermal annealing is negligible. The black and red dashed curves are corresponding to depth-integral curves of the surface-depth profiles measured by SIMS and calculated by SRIM. Both integrated curves are saturated (~99.5%) at a certain depth (276 nm), which is distant from the peak by about twice the standard deviation of the Gaussian depth-distribution. In the present study, this depth is called as “implanted depth”. We calculated the implanted depths under other conditions in Table S1 and confirmed that the implanted depth is smaller than the film thickness in any cases. This indicates that in our experiments, our electrical measurements were done within the nanostructure films, not in the substrates.

Assuming a constant activation rate of implanted ions, the carrier concentration n is proportional to dopant concentration. Then, n has the same surface-depth profile shape as that of dopant concentration. Therefore, we defined that the carrier conduction layer thickness, L as the aforementioned implanted depth. The sheet carrier concentration, n_S is obtained by Hall measurements. We defined the average carrier concentration, n_A by setting the product of n_A and carrier conduction layer thickness to be the n_S value as shown in Fig. S1(b). The n surface-depth profile can be also determined because the integral of n surface-depth profile is equivalent to n_S.

The activation rate can also be obtained by the ratio between the n_S and dopant ion dose (=the integral of dopant surface-depth profiles). In the main text, the n_A represents the carrier concentration of the samples.

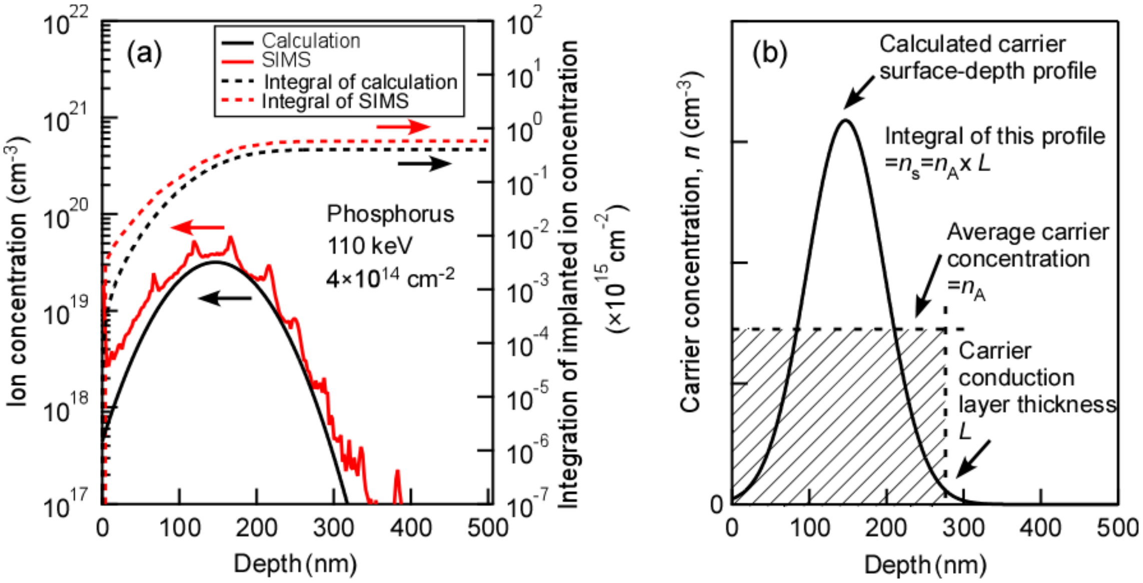

Figure S1 Dopant surface-depth profiles and surface-depth profiles of carrier concentration

(a) SIMS P surface-depth profile of 8-nm NDs/376-ML Si sample (red solid curve). P surface-depth profile calculated by SRIM program exhibits Gaussian depth-distribution (black solid curve). The black and red dashed curves are corresponding to depth-integral curves of the surface-depth profiles measured by SIMS and calculated by SRIM, respectively. (b) The schematic of surface-depth profile of carrier concentration and carrier conduction layer thickness.

III. Validity of n_A in terms of electrical conductivity and Seebeck coefficient

In chapter II, we defined L and n_A. In this chapter, we confirmed the validity of the n_A definition in terms of electrical conductivity σ, and Seebeck coefficient, S.

The carrier mobility μ and σ have the n-dependence: namely depth-dependence. We calculated surface-depth profile of σ using μ with n-dependence of bulk Si as shown in Fig. S2(a). This is a similar surface-depth profile to that of n. We compared the difference of the L obtained from n surface-depth profile (chapter II) and the saturated depth in σ surface-depth profile, at which integrated curve of σ surface-depth profile is saturated (~99.5%). The difference is very small (usually ~6-7%). This indicates that the depth-dependence of μ and σ does not influence the determination of the conduction layer thickness.

To show the validity of this definition in terms of electrical conductivity σ, and Seebeck coefficient, S experimentally, we implanted dopant atoms into non-doped bulk Si for doping. Then, we evaluated Hall electron mobility μ_{He}, Seebeck coefficient S, and electron concentration n of the implanted bulk Si using the definition of the aforementioned L and n_A. At the various carrier concentrations, these values exhibited almost the same values as those of the conventional (non-implanted) uniformly-doped bulk Si [S1-S3] as shown in Fig. 3b and 3d. This proved that we can evaluate the μ_{He}, S, σ, and n_A under this definition.

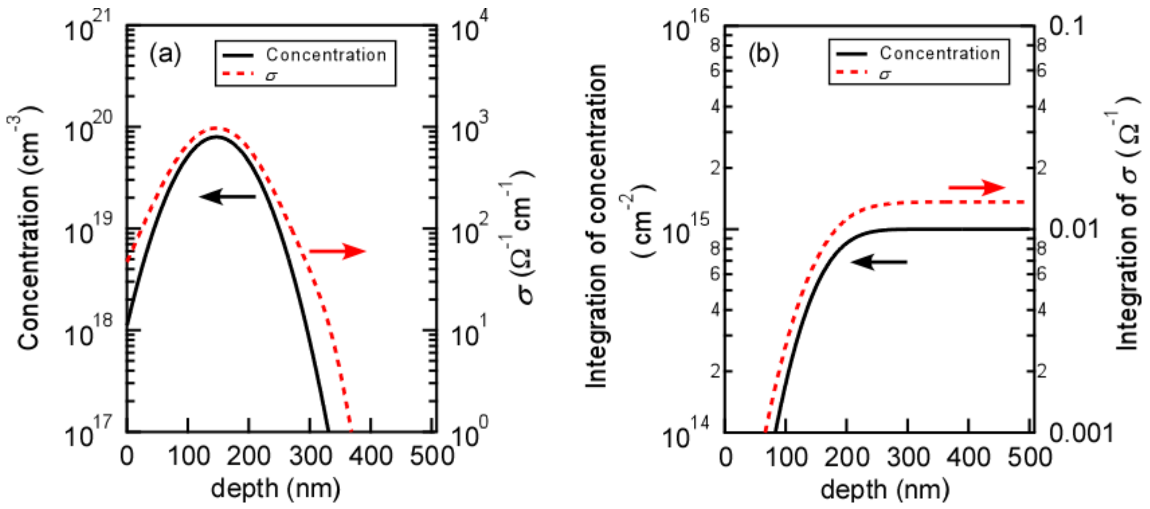

Figure S2 Dopant surface-depth profiles of carrier concentration and electrical conductivity

(a) The calculated surface-depth profiles of n and σ. (P-ion 110 keV). (b) The integrated curves of (a)

IV Structural characters of the nanoarchitecture

Our nanoarchitecture has the following structural characters as shown in Figs. S3 and S4. Figure S3 shows the random position of Ge NDs in the nanoarchitecture with thin Si layer in one cycle structure, indicating reduction of structural anisotropy. Figure S4 shows ansyses of the fast Fourier transformation (FFT) patterns around Ge NDs in the high-angle annular dark-field scanning transmission electron microscopy (HAADF-STEM) image of the stacked structure. The lattice spacing analyses of FFT patterns suggest that Ge NDs, with 4% lattice mismatch, are fully relaxed, as reported in our previous work [S5], and that Si layer is elastically strain-relaxed just above Ge NDs within a typical distance of ~ 1 nm. The same strain distribution was also observed in the samples after carrier doping of P, indicating carrier doping does not affect the structural properties of nanoarchitecture. This is applicable to the case of carrier doping of B because of the same dose. Also, strain effect should be considered when we discuss the conduction band offset at Si/Ge NDs and it complicates the energy band diagram [S6]. However, we considered that this effect could be negligible in our case of non-strained Si layer except for the ultrasmall regions (<~1-nm distance from Ge NDs).

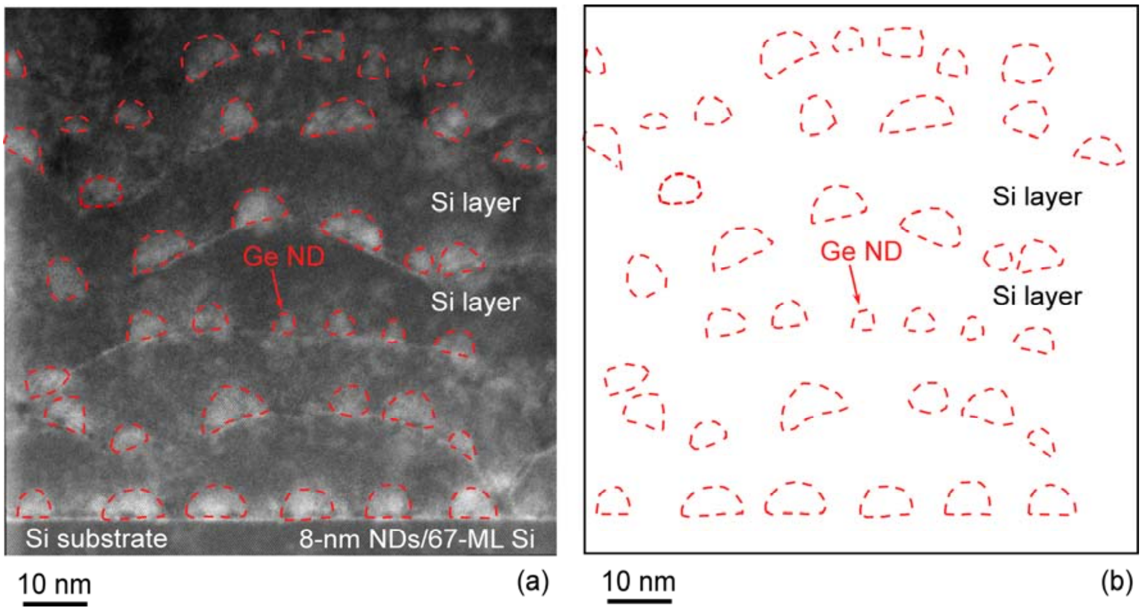

Figure S3 HAADF-STEM image of an isotropic "thin-Si layer" nanoarchitecture.
(a) The HAADF-STEM image of the stacked structure with thin Si layer. (b) The schematic image showing isotropic (random) Ge ND distribution in the nanoarchitecture with thin Si layer in one cycle structure.

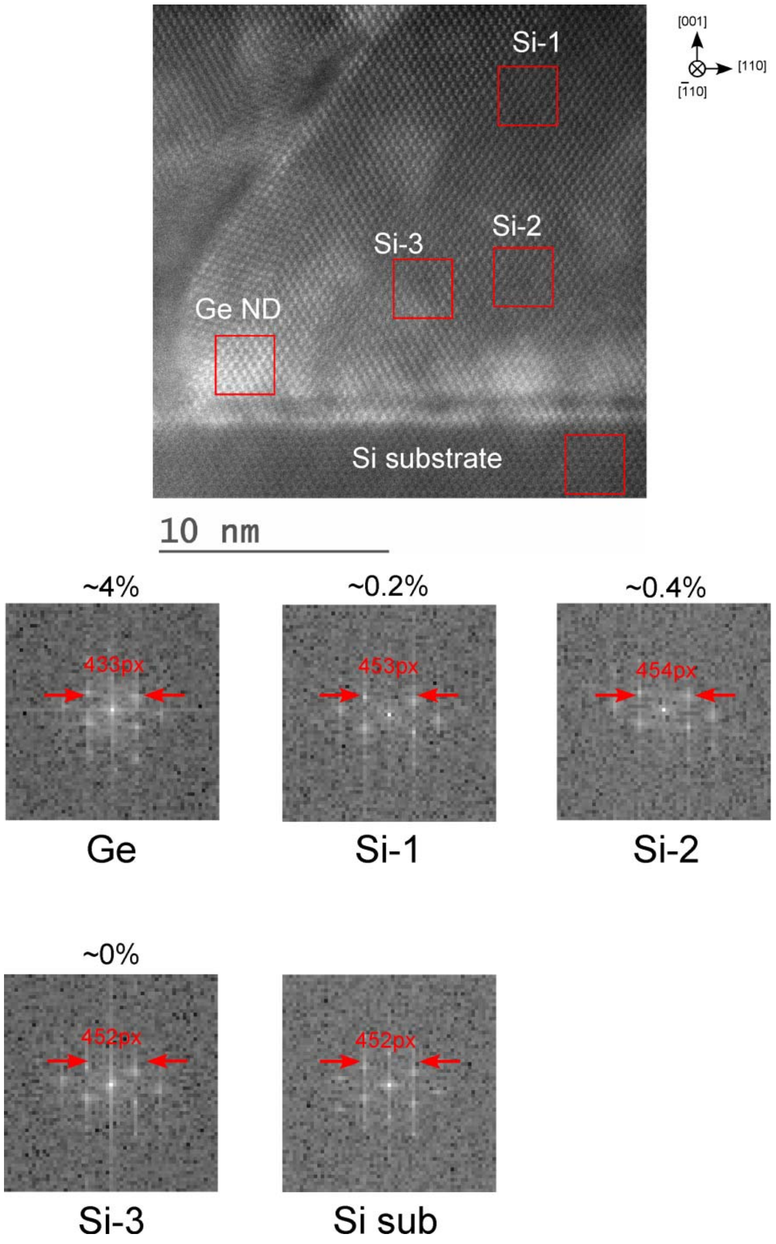

Figure S4. Structural analyses around the Ge NDs layer

HAADF-STEM image of the stacked structure and the fast Fourier transformation (FFT) patterns around Ge NDs. The values denoted above FFT patterns were the difference (%) of lattice spacing from that of Si substrates, where positive sign means larger lattice spacing than that of Si substrate. The error in FFT pattern analysis is ~3px corresponding to ~0.66% error.

References

[S1]. D. K. Schroder: *Semiconductor Material and Device Characterization, 3rd Edtition,* Wiley, Hoboken, 2006.

[S2]. Y. Ohishi, J. Xie, Y. Miyazaki, Y. Aikebaier, H. Muta1, K. Kurosaki, S. Yamanaka, N. Uchida, and T. Tada, *Jpn. J. Appl. Phys.* **54**, 071301 (2015).

[S3]. O. Yamashita, and N. Sadatomi, *Jpn. J. Appl. Phys.* **38**, 6394 (1999).

[S4]. S. M. Sze, *Physics of Semiconductor Devices, 2nd Edition*, Wiley, New York, 1981.

[S5]. Y. Nakamura, A. Murayama, and M. Ichikawa, *Cryst. Growth Des*. **11**, 3301 (2011).

[S6]. C. G. Van de Walle, R. M . Martin, *Phys. Rev. B* **34**, 5621 (1986).
